# From Pathogen to Toxin: The Revolutionary Work of Dr. Sambhu Nath De in Understanding Cholera

**DOI:** 10.7759/cureus.68414

**Published:** 2024-09-01

**Authors:** Alisha Handa, Sonali G Choudhari, Abhay Gaidhane

**Affiliations:** 1 Department of Community Medicine, School of Epidemiology and Public Health, Jawaharlal Nehru Medical College, Datta Meghe Institute of Higher Education and Research, Wardha, IND

**Keywords:** cholera toxin, intestinal loop model, oral rehydration therapy, public health, historical vignettes

## Abstract

In the history of medical science and public health in India, the groundbreaking research on cholera toxin by Dr. Sambhu Nath De presents a pivotal moment. This review article dives into Dr. De's influential contributions to the understanding of cholera pathogenesis, his discovery of the cholera toxin, and its implications for the treatment and prevention of diseases. By defining the process via which vibrio cholera causes severe diarrhea and dehydration, Dr. De worked toward paving the way for the development of effective rehydration therapies and preventive strategies that have helped save innumerable lives all over the world. This article also reviews the more significant impact of Dr. De’s findings in the field of public health and its shaping and use of modern approaches to infectious control. This article aims to honor the enduring legacy and contributions to public health reform by Dr. De.

## Introduction and background

**Figure 1:**
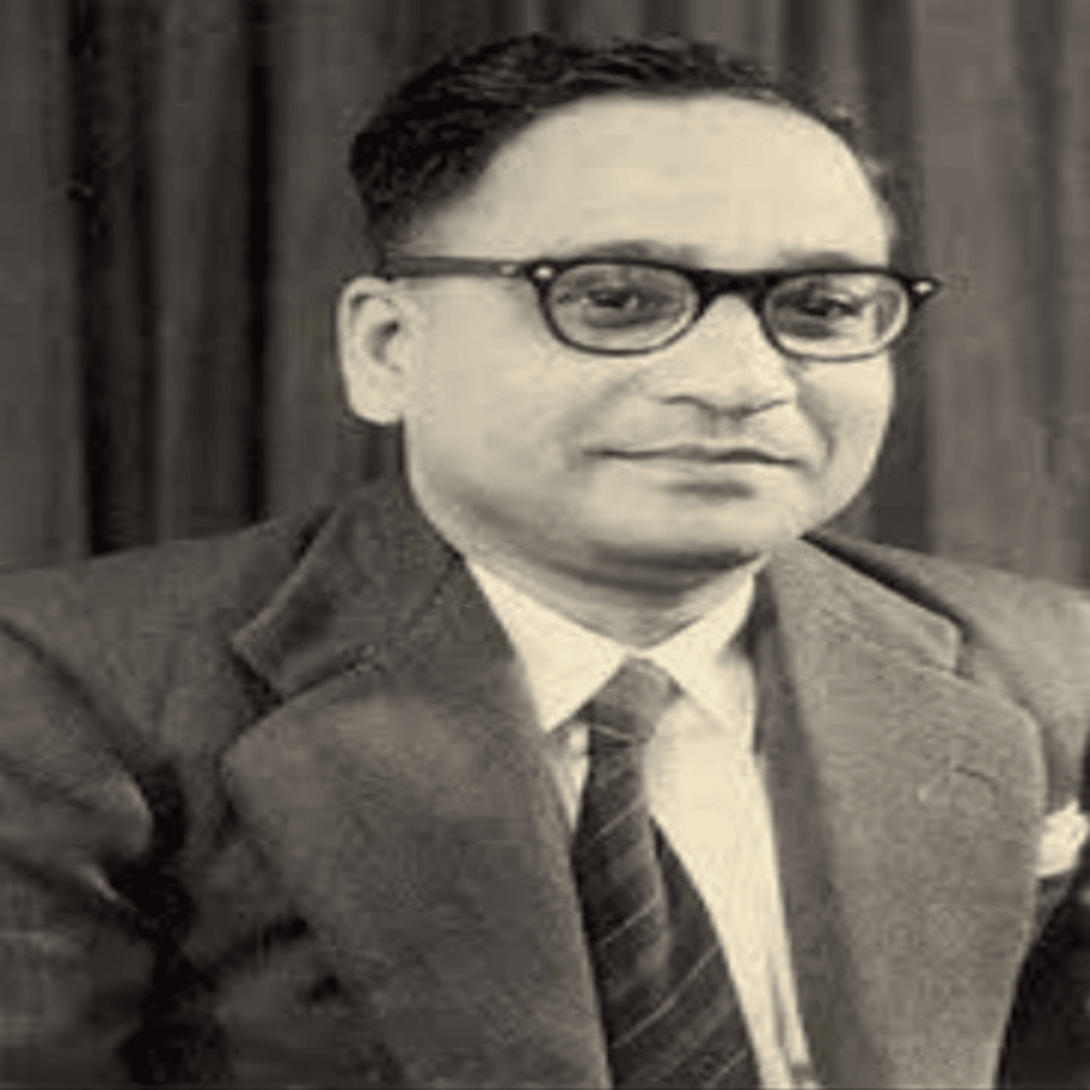
Prof. Sambhu Nath De, an Indian Bengali medical scientist Image courtesy of Wikimedia Commons (public domain) Source: [3]

Sambhu Nath De (Figure 1), an Indian researcher and medical expert, was born on February 1, 1915 and died on April 15, 1985. The bacterium causing cholera was found in 1884, but the scientists failed to identify the appropriate antidote for it. The scientists working on finding the cholera bacterium were doing so in nations where there was no cholera epidemic, considered as the reason by Koch. On the other hand, no scientists from India were working on cholera where it was widespread and often found in epidemic forms. On the other side, De did not believe in Koch's poison theory because he thought that it was the exotoxin that killed the victims. Thus, this review article highlights the significant contributions by De to medicine with the identification of the cholera toxin, the cholera animal model, and the successful illustration of the *Vibrio cholerae* bacterium's path of transmission [1].

In 1959, Sambhu Nath De illustrated the cause of cholera, which was the isolation of cholera toxin. The cell surface of the bacterium has several pilli and a flagellum at one of the poles. The bacterium undergoes fermentative and respiratory metabolism. The serogroups responsible for cholera outbreaks are O1NCBI: *Vibrio cholerae* O1 and O139NCBI: *Vibrio cholerae* O139. The infection is spread essentially through drinking contaminated water, so it is associated with hygiene and sanitation. When ingested, it invades the mucosa of the intestine of the host and causes vomiting and diarrhea within several hours to two to three days of ingestion. *V. cholerae* has two circular DNAs. One of them is a protein that causes watery diarrhea (rice-water stools) [2].

## Review

Life and career trajectory

Sambhu Nath De was born on the Ganga's western bank in the village of Garibati. Floods destroyed the family's prosperous business, leaving Dashurathi De and Chattesweri, his parents, bankrupt. The family was part of a huge joint family. Later, in order to support the family, his father operated a modest business. As the only educated member of the family, his uncle Ashutosh De gave special attention to Dr. De's academic career, enabling him to pass his 10th-grade exams with distinction from Garbati High School and go on to Hooghly Mohsin College on scholarship.

De graduated with a Diploma in Tropical Medicine in 1942 after completing his M.B. at Calcutta Medical College in 1939. Under the supervision of Sir Roy Camero, he also earned his PhD in pathology from the University College Hospital Medical College in London in 1949. Following his return, he focused on the pathophysiology of cholera and published the results. His future career was significantly shaped by pathology and bacteriology professor M N De, who was deeply impressed by Dr. De's demeanor and performance, and De was married to his daughter, Torubala [3]. In 1955, De traveled to England with funds from the Nuffield Foundation and the Royal Society to give a research report on* V. cholerae* and *E. coli* to the Pathological Society of Great Britain. His creative work was well-received [4].

He started working at Nil Ratan Sarkar Medical College after returning to India, and he later became the Head of the Department of Pathology and Bacteriology at Calcutta Medical College, where he remained until his retirement.

During this period, the city of Calcutta was threated by the cholera disease. This made him dig deeper into the disease, and thus, he pioneered his work in the field of bacteriology [5].

The medical man of the Blue Death

Known by the name "Blue Death," or Asiatic cholera, this extremely contagious disease most likely started in India and caused three significant epidemics in West Bengal in the 1800s before migrating throughout the globe and killing millions of people.

India was responsible for 86,997 deaths (or 49.34%) out of 1,76,307 cases reported in 1950. Although there were still 12,947 deaths 15 years later, the fatality rate dropped to 29.9%, according to The Indian Express. De's work was crucial for the world at large and for the Indian subcontinent [6].

Robert Koch discovered the cause of cholera, but according to him, this bacterium enters the person's circulatory system and affects it. De postulated that the cells lining the small intestine were the primary target of the cholera bacillus.

The experiment

Dr. De put rabbits under anesthesia and put *V. cholerae* into their intestinal lumens to demonstrate this. The rabbits passed away a few days later rather than having diarrhea. According to De, the large caecum of the mice he employed for the experiment often held semisolid material, but on this occasion, it was full of semiliquid feces, which allowed for the recovery of *V. cholerae* [7]. According to research by De et al. (1951), injecting *V. cholerae* intraperitoneally into rabbits killed and cleaned them, resulting in an accumulation of fluid in the peritoneal cavity and the symptoms of severe watery diarrhea [8].

He continued to do research that gave rise to the idea that *E. coli* may produce enterotoxins, a theory that is now firmly established as enterotoxigenic *E. coli *[9]. He also discovered that exotoxin is the toxin produced by this bacterium. He wanted to continue his research on exotoxin further but could not find more resources in India back then [10].

De discovered the cholera toxin about 75 years after Robert Koch first cultivated *V. cholerae*, then known as comma bacillus, in Calcutta and 105 years since Italian anatomist Filippo Pacini examined the cholera causative agent under a microscope and published the groundbreaking epidemiologic studies that explained the disease's waterborne transmission. 

Before that, the consensus was that "miasma," or unsavory or harmful vapors coming from sewers, was the source of the illness [11].

Recognition and impact

Dr. De’s demonstration that dehydration was a sufficient cause of death for cholera and other diarrheal diseases significantly advanced our understanding of these illnesses. Nevertheless, despite being often nominated for the Nobel Prize, the physician Sambhu Nath De (1915-1985) did not win the honors [12]. However, his contributions were later acknowledged internationally, including nominations for the Nobel Prize. After some time, De's discovery of a diarrheagenic exotoxin served as the basis for contemporary cholera research, immunization, and therapy. In addition, a scientific breakthrough led to *V. cholerae* being recognized as the enteric pathogen with the most comprehensive understanding at the molecular level. The effectiveness of oral rehydration, which has been called one of the most significant medical developments of the 20th century, was explained, and the scientific foundation for giving vaccinations orally in order to develop mucosal immunity was developed [13]. Even though the revelation was highly anticipated rather than revolutionary, De's novel pathogenesis model had an unexpectedly small immediate effect. A few years of delay later, it was acknowledged, and a new wave of researchers elevated cholera from scientific obscurity to paradigm status. Even though the discoveries of sodium/glucose co-transport and cholera toxin solidified the scientific basis for oral rehydration's efficiency and tolerance, the development of therapy was likewise postponed [14].

“The majority of cholera experts worldwide agree, in my opinion, that your effort was extremely important” were the words of van Heyningen for Professor De.

De's study initiated a new way of looking at the intricate process that looks like diarrhea, and as a result, it had a long-lasting effect on the history of cholera research and the history of cellular physiology and biochemistry. The finding of Shiga and Shiga-like toxins, as well as the entire families of labile toxins from enterotoxigenic *E. Coli *that cause diarrhea, was also made possible by De's work. De's finding gave immunologists additional directions to pursue, mainly when it came to examining the immune responses to the toxin and developing an antitoxin vaccination [15].

It has been 50 years since De discovered the cholera toxin, and it has been 128 years since Koch isolated pure cultures of the comma bacillus for the first time. Both events occurred in 2009. Even though we have learned a great deal about *V. cholerae* over the past 128 years, including the whole genomes of 24 isolates, the cholera epidemic nevertheless persists in many parts of the world [16].

"De's clinical observations led him to the audacious conclusion that the cholera toxin might kill a person "merely" by inducing the release of water into the colon and that dehydration was a sufficient cause of cholera pathology," as Nobel Laureate Prof. Joshua Lederberg put it. Therefore, De's discovery of the cholera toxin should be directly attributed to the development of oral rehydration therapy, which has saved countless lives by replacing the vast fluid loss in cholera patients. His research on exotoxins helped to develop a series of cholera and enterotoxigenic *E. coli* (included in the modern perspective of diseases caused by toxin-producing bacteria) and set the stage for the purification of Cholera and heat-labile enterotoxins produced by *V. cholerae* and *E. coli*, respectively [1].

Three of De’s works are noteworthy: the ligated intestinal loop method; the ileal loop model, which showed the connection between cholera-like diarrhea and a strain of *E. coli* that produced toxins; and, finally, and perhaps most significantly, the 1959 discovery of bacteria-free culture filtrate of* V. cholerae* that triggered a particular cellular response. In the years that followed, detailed reports on the endotoxin and exotoxins from the culture filtrate were made [4].

Life and retirement

In 1973, De withdrew from the medical college at the age of 58. He did not indicate interest in the directorship at the college, which would have eventually led to the director of health services, nor did he ask for the customary two-year extension. The fact that he was unable to complete his studies as he had desired owing to circumstances beyond his control frustrated him. It made him lose interest in teaching and the administrative field. Before entering private practice, he established a clinical pathology laboratory at his house to keep himself occupied and physically active. A few years after he had retired, in 1978, he received a call from the Nobel Foundation inviting him to participate in the 43rd Nobel Symposium on Cholera and related subjects. De discussed his research on the serotyping of *E. coli *during his presentation at this symposium.

De was different from the kind who thrived in big crowds, workshops, and conventions. He found happiness in a close-knit group of friends and business associates who were like family. A friend who would check on his family and provide gifts for the kids was the nature of Dr. Despite never having practiced medicine, he helped numerous patients with guidance and had a remarkable clinical diagnostic eye. On April 15, 1985, he passed away. He received a letter from S. Arunachalam, Editor of the Indian Journal of Technology, a few hours before he passed away while he was unconscious. The letter asked him to contact Eugene Garfield, Editor of Current Contents, as he was curious about his background and professional accomplishments [3].

"Discoveries that are presented in the scientific literature are often presented in a way that provides little to no insight into the actual processes involved." S N De referenced Arne Tiselius in his introductory remarks at the 43rd Nobel Symposium on "Cholera and Related Diarrhoeas" in Commentary, Vol. 11, N. 26 (1978).

## Conclusions

The innovative discovery of Sambhu Nath De transformed the understanding of cholera, which marked a milestone in the field of public health and microbiology. The discovery by De that the cholera bacterium produces a potent toxin that causes severe dehydration and diarrhea in cholera patients is groundbreaking. This discovery was a pivotal shift in focusing the treatment from bacteria to neutralizing the toxin, leading to the development of oral rehydration therapy. His work exemplifies the importance of research methods and the relentless pursuit of scientific truth. Thus, his legacy goes beyond cholera research and influences the fields of bacteriology and public health. His story underscores the importance of perseverance and determination against all odds and says so much about how one individual can impact global health.
